# Supplementary material for: Identification of a Novel CLPX Variant in a Mixed-Breed Dog with Anemia and Spinocerebellar Ataxia
Source: Genes (Basel). 2025 Nov 10;16(11):1359. doi: 10.3390/genes16111359 (PMC12652279; doi:10.3390/genes16111359)
Supplement: Supplementary file 1 [file genes-16-01359-s001.zip › Supplemental information_Genes_Updated_10.22.25.pdf]

Table S1: Complete blood count results from the case dog.

	Value	Unit	Reference range
RBC	**1.84**	10^6/μL	5.40 – 8.40
Hemoglobin	**4.8**	g/dL	12.0 – 18.0
Hematocrit	**14.2**	%	35.0 – 54.0
MCV	**77.5**	fL	62.0 – 77.0
MCHC	33.4	g/dL	32.0 – 37.0
CHCM	35.2	g/dL	31.6 – 35.3
RDW	14	%	12.0 – 14.0
Platelets	**148**	10^3/μL	220 - 600
WBC	9.7	10^3/μL	8.0 – 14.5
Abs Seg	8.9	10^3/μL	3.0 – 11.5
Abs Lymph	**0.68**	10^3/μL	1.0 – 4.8
Abs Mono	**2.0**	10^3/μL	0.1 – 1.4
% Retic	<0.5	%	<0.5
Abs Retic	**2.5**	10^3/μL	<92,000 /μL: inadequate or no regeneration

Bolded values are abnormal. RBC: Red Blood Count; MCV: Mean Corpuscular Volume; CHCM: Cell Hemoglobin Concentration Mean; RDW: Red Cell Distribution Width; WBC: White Blood Count. Abs: Absolute; Retic: Reticulocytes.

Table S2. Separately submitted as a Microsoft Excel workbook with multiple sheets. Variants detected by whole genome sequencing and private variant analysis in the case dog affected by spinocerebellar ataxia (sheet 1). Private variants were obtained by filtering out all variants that were present at least once in 748 control genomes. This resulted in 3,864 homozygous private variants (sheet 2) and 26,911 heterozygous private variants (sheet 3). Variants predicted to have a “high” or “moderate” effect on resulting transcript were considered to be protein-altering and retained. This resulted in 17 protein-altering homozygous private variants (sheet 4) and 175 protein-altering heterozygous private variants (sheet 5). (Note: variants listed multiple times if they have predicted effects on more than one transcript). One homozygous private variant and 2 heterozygous private variants were in directly related functional candidate genes for similar phenotypes in other species (VarElect score ≥ 10; sheet 6).

Table S3: Protein pathogenicity predictive software results for the two heterozygous variants with VarElect scores ≥ 10. % values indicate the confidence level in that result; MutPred2 provides a score, where anything < 0.5 is considered tolerated. Nine of the programs predicted the *ERCC4* variant to be neutral and not pathogenic. The *vWF* variant, even when ranked as “deleterious”, was lower in confidence.

**Pathogenicity Prediction Software**	**ERCC4:p.Val81Ile**	**VWF:p.Arg1399His**
PredictSNP	Neutral (83%)	Deleterious (51%)
MAPP	Neutral (85%)	-
PhD-SNP	Neutral (83%)	Neutral (45%)
PolyPhen-1	Neutral (67%)	Neutral (67%)
PolyPhen-2	Neutral (72%)	Deleterious (51%)
SIFT	Neutral (71%)	Deleterious (53%)
SNAP	Neutral (77%)	Deleterious (56%)
PANTHER	Neutral (64%)	-
MutPred2	Tolerated (0.099)	Tolerated (0.469)

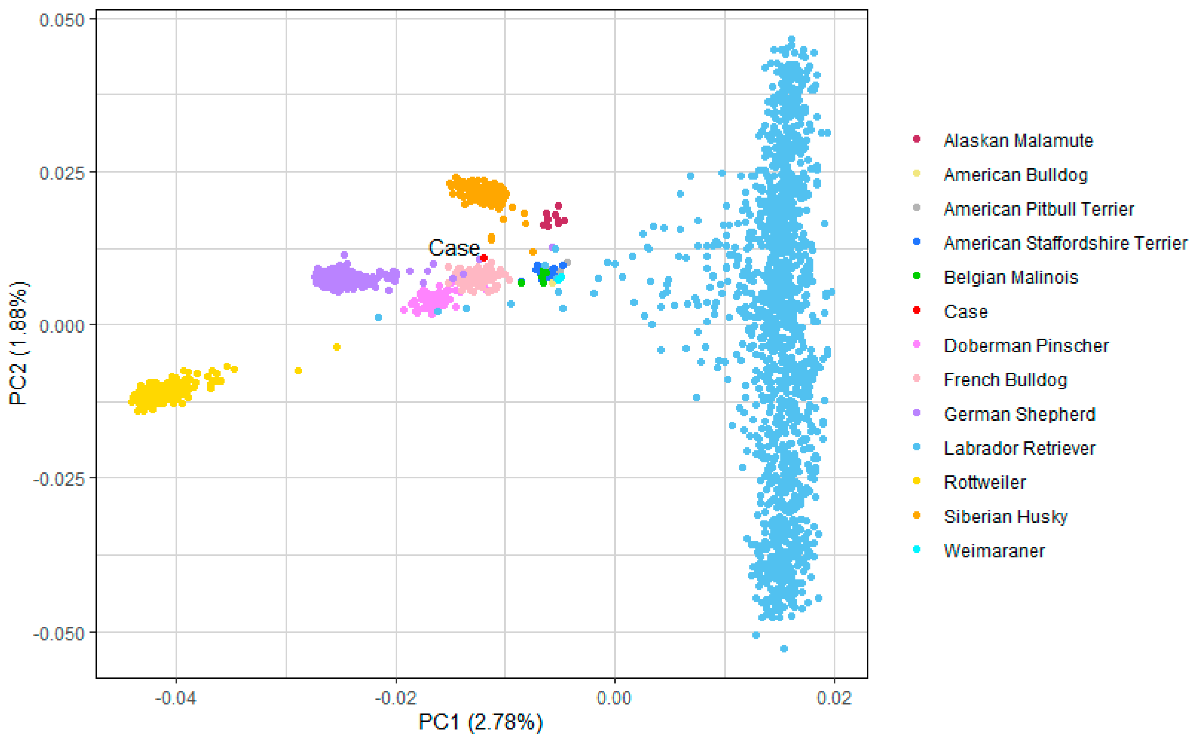

Figure S1: Principal component analysis (PCA) of the genetic make-up of the case and twelve different dog breeds: Alaskan Malamute (n = 9), American Bulldog (n = 2), American Pitbull Terrier (n = 6), American Staffordshire Terrier (n = 11), Belgian Malinois (n = 8), Case (n = 1), Doberman Pinscher (n = 77), French Bulldog (n = 101), German Shepherd (n = 190), Labrador Retriever (n = 1434), Rottweiler (n = 293), Siberian Husky (n = 140), and Weimaraner (n = 2).

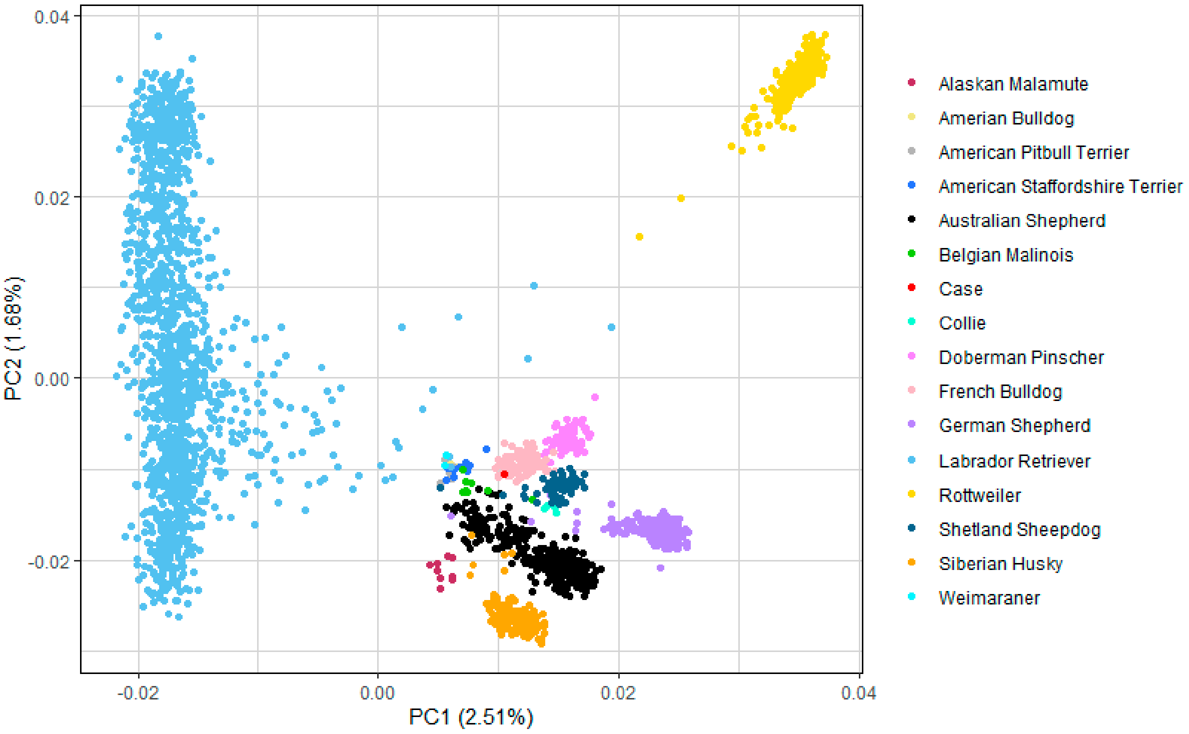

Figure S2: Principal component analysis (PCA) of the genetic make-up of the case and fifteen different dog breeds: Alaskan Malamute (n = 9), American Bulldog (n = 2), American Pitbull Terrier (n = 6), American Staffordshire Terrier (n = 11), Australian Shepherd (n = 294), Belgian Malinois (n = 8), Case (n = 1), Collie (n = 5), Doberman Pinscher (n = 77), French Bulldog (n = 101), German Shepherd (n = 190), Labrador Retriever (n = 1434), Rottweiler (n = 293), Shetland Sheepdog (n = 63), Siberian Husky (n = 140), and Weimaraner (n = 2).

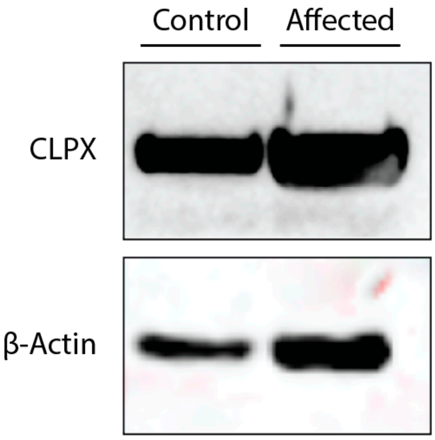

Figure S3: Representative western blot results of CLPX in cardiac tissues from an unaffected control dog and the spinocerebellar affected dog. Each row is a cropped image showing the results generated when the membrane was probed with the primary antibody listed on the left. In this experiment, β-actin was used as the loading control.

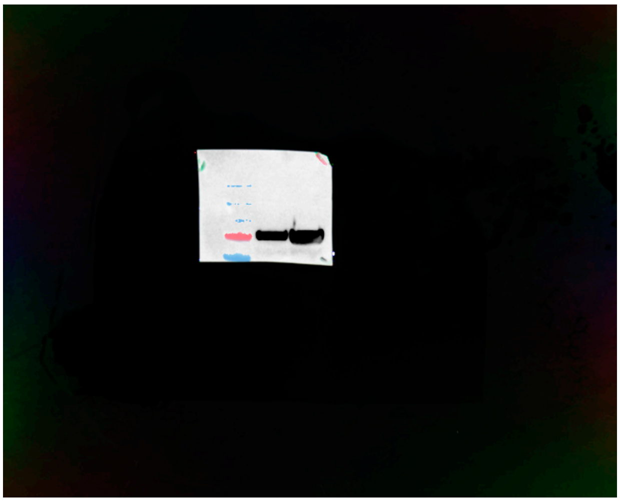

Figure S4: Original untrimmed image of the western blot analysis performed to detect CLPX in the cardiac muscle tissue from the affected dog and an unaffected control Border Collie. Lane 1: PageRuler Plus Prestained Protein Ladder (26619; ThermoFisher Scientific). Lane 2: approximately 50μg of protein from whole-tissue lysate of unaffected control Border Collie. Lane 3: approximately 50μg of protein from whole-tissue lysate of the affected dog

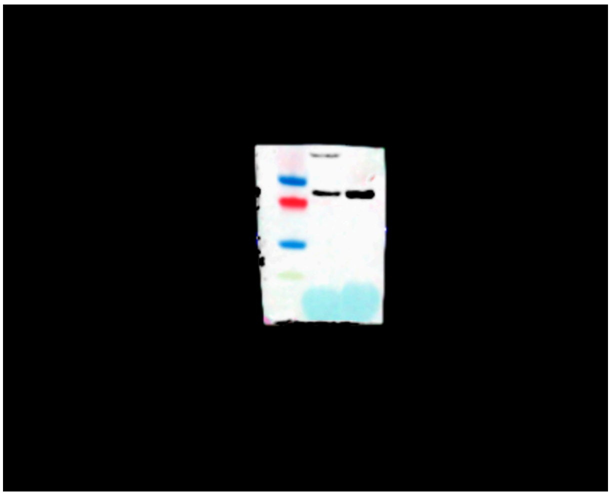

Figure S5: Original untrimmed image of the Western blot analysis performed to detect β-actin in the cardiac muscle tissue from the affected dog and an unaffected control Border Collie. Lane 1: PageRuler Plus Prestained Protein Ladder (26619; ThermoFisher Scientific). Lane 2: approximately 50μg of protein from whole-tissue lysate of unaffected control Border Collie. Lane 3: approximately 50μg of protein from whole-tissue lysate of the affected dog
